# Acceptability of *My Ten*: a physical activity intervention for family caregivers of people with advanced cancer

**DOI:** 10.1186/s12904-026-02309-4

**Published:** 2026-09-14

**Authors:** Jamie L. Penner, Sylvie Lambert, S. Robin Cohen

**Affiliations:** 1 College of Nursing, Rady Faculty of Health Sciences, University of Manitoba, 89 Curry Place, Winnipeg, MB R3T 2N2 Canada; 2 Ingram School of Nursing, McGill University, 680 Sherbrooke West, Suite 1800, Montreal, QC H3A 2M7 Canada; 3 Departments of Oncology and Medicine, McGill University; Investigator, Lady Davis Research Institute of the Jewish General Hospital, 3755 Côte-Sainte-Catherine Road, Montreal, QC H3T 1E2 Canada

**Keywords:** Caregivers, Physical activity, Neoplasms, Health, Self-care, Health promotion, Exercise, Palliative care

## Abstract

**Background:**

The caregiving experience can have a significant impact on family caregivers’ (caregivers) health and well-being, particularly those of patients with advanced cancer. Interventions to support caregivers through health-promoting strategies are needed. The physical and psychological benefits of physical activity (PA) are well documented, yet the value of PA for caregivers of people with advanced cancer is not well understood. To address this gap, a home-based individually tailored PA intervention was designed with the aim of increasing caregivers’ positive affect and health and decreasing their negative affect and fatigue. The objective of this study was to explore the acceptability of the intervention for caregivers.

**Methods:**

A descriptive exploratory qualitative research design was used. Caregivers (*N* = 10) were recruited to participate in the intervention for 6 weeks and provided weekly qualitative feedback on its structure and process. A follow-up individual interview was conducted with caregivers to solicit their overall experience with the intervention. Data were analyzed using content analysis.

**Results:**

Eight of 10 caregivers completed the intervention. Caregivers deemed the PA intervention to be acceptable. No modifications were necessary other than slight changes in formatting to the PA log. Caregivers were appreciative of attention to and support of their own self-care. A home-based PA program allowed them to take breaks from caregiving responsibilities while staying near the patient. Individually tailored PA, goal setting and self-monitoring were helpful for caregivers to engage in and adhere to their PA plans. In addition, weekly telephone calls for feedback and support helped to problem solve any barriers and provided motivation. Given that caregivers often chose to engage in smaller, more frequent bouts of PA, the intervention was named *My Ten*.

**Conclusions:**

The *My Ten* PA intervention is acceptable to caregivers of those with advanced cancer, helping them to engage in and adhere to PA as a health-promoting strategy.

**Supplementary Information:**

The online version contains supplementary material available at https://doi.org/10.1186/s12904-026-02309-4.

## Background

Family caregivers of individuals living with advanced cancer (caregivers) are faced with various physical, emotional, and financial demands in their caregiving role whilst dealing with their own emotional responses to the patient’s diagnosis and the uncertainties related to living with advanced illness [1–4]. At the same time, caregiving responsibilities, carrying out multiple roles, and often being unable to leave the patient unattended, impose significant disruptions in caregivers’ routines, and restrictions on their time, freedom, and ability to participate in social and health-related activities such as maintaining a healthy diet, engaging in physical activity (PA), and getting adequate rest [4–7]. While aspects of being a caregiver can be rewarding, high levels of fatigue and negative changes to caregivers’ psychological and physical health and wellbeing have been well documented [8, 9], prompting the need for supportive interventions to be developed.

To date, caregiver interventions have focused mainly on increasing caregivers’ knowledge and skills to prepare them for and support them with the provision of care [10–12]. While an increasing number of interventions to address caregivers’ self-care have also been developed, these are mostly psychoeducational strategies aimed at promoting emotional coping and problem solving [13]. Fewer interventions have been developed to support caregivers of those with advanced cancer using behavioural health-promoting strategies [10, 13]. Effective, evidence-based interventions targeting health-promotion strategies are needed to support caregivers so that they may keep from becoming ill themselves and continue to provide care for their family member, if they desire.

Physical activity (PA) has long been considered an important health-promoting behaviour, and the physical and psychological benefits of PA are well documented [14, 15]. While guidelines exist endorsing specific levels of PA to achieve optimal health benefits [16], most caregivers are not engaging in PA at these recommended levels [17–21]. Moreover, the PA they are engaging in often declines after becoming a caregiver [6, 7, 22, 23]. There is increasing evidence that engaging in even small amounts of PA, particularly when increasing from no, or little, activity has marked health benefits [24]. In addition, PA can be tailored to the needs, preferences, and circumstances of individuals. Therefore, helping caregivers to increase, or simply maintain, their engagement in PA considering their caregiving situation is an important health-promoting strategy.

In the context of cancer, PA interventions for caregivers [17, 25] or caregiver-patient dyads [26–30] have recently been developed. However, to date, only one behavioural health-promotion intervention has been reported specifically for caregivers of people with advanced cancer [31]. While PA is included as one of several health-promoting behaviours that is monitored and that caregivers are educated about in this multicomponent intervention, it is unclear if all caregivers are required to set goals related specifically to PA or how they engaged in it. Rigorously designing an intervention explicitly for caregivers of those with advanced cancer is important, as the experience and needs of caregivers of those with advanced cancer is different than those earlier in the cancer trajectory [32, 33]. Caregivers of those with advanced cancer often experience significant burden owing to complex care responsibilities and greater dependency of the patient [34, 35] and often report needing a mental break from their caregiving responsibilities [36, 37]. As the patient’s disease progresses and their condition worsens, the demands on caregivers increase, as do their levels of fatigue and psychological distress [32, 38–40], reinforcing the importance of an individualized, flexible PA intervention that is integrated into caregivers’ daily lives rather than imposing an additional obligation.

Caregiver-focused interventions align with palliative care models that recognize caregivers as individuals with their own health needs, not solely as providers of patient support [41, 42]. Caregiver-focused and dyadic interventions are not competing approaches but rather address different intervention targets. Dyadic interventions may target individual patient outcomes, individual caregiver outcomes, and/or shared patient-caregiver outcomes, such as communication, relationship functioning, or mutual coping [43], whereas caregiver-focused interventions are designed to improve caregivers’ self-care behaviours [44] and allow the intervention to be tailored to caregivers’ needs without being constrained by the patient’s functional status [32, 39], symptom burden [33, 45], or ability to participate [46, 47]. This is particularly relevant in the context of advanced cancer, where progressive functional decline and an unpredictable illness trajectory may limit opportunities for sustained dyadic participation in PA. Interventions tailored to caregivers’ changing circumstances, preferences, and available time may reduce barriers to participation and support ongoing engagement, even as caregiving demands evolve.

An empirically and theoretically based PA intervention was designed for caregivers of people with advanced cancer with the aim to increase or maintain their PA and thereby, ultimately, increase positive affect, decrease negative affect and fatigue, and improve their self-rated health. Given that PA interventions for these caregivers are newly emerging, the Health Promotion Model [48] served as the framework to supporting PA engagement. Embedded in the Health Promotion Model is a collaborative relationship between the intervener and the caregiver. The model focuses on behaviour-specific cognitions and affect (perceived benefits of action; perceived barriers to action; perceived self-efficacy; activity-related affect; interpersonal influences; and situational influences) that can be explored through motivational interviewing, to inform individualized plans and promote engagement in PA. It also identifies commitment to PA, characterized by an intent and a detailed plan of action, as influencing engagement in the desired behaviour. Finally, the Health Promotion Model outlines ongoing feedback as an important component to determine if the plan worked and make any necessary adjustments. The behavioural outcome is engagement in the targeted health promoting behaviour.

Assessing how well the intervention will be received by the target population (its acceptability), is an important step in the early stages of intervention development [49]. Feedback on participants’ attitudes towards, experiences with, and perceptions of the intervention are recognized as a valuable indicator of its acceptability [50] and can guide the development and/or refinement of the intervention and enhance its favorableness to the target population. In addition, behaviours such as adherence to the intervention and attrition can provide helpful insight into an intervention’s acceptability [51]. An intervention that is acceptable enhances its uptake, implementation, adherence, and effects on the desired outcomes [51]. Therefore, this study evaluated the acceptability of a newly designed PA intervention for caregivers of people with advanced cancer, including perceived relevance and usefulness, attrition, and adherence to PA plans.

## Methods

### Design

A descriptive exploratory qualitative research design [52, 53] was used. Qualitative descriptive studies explore poorly understood issues while keeping findings close to participants’ perspectives. This approach can guide intervention refinement by identifying salient and unanticipated factors affecting uptake, making it well suited to assessing acceptability [49, 53–55]. Using a consultative, deductive approach, caregivers trialed an abbreviated PA intervention and provided feedback on its relevance, usefulness, benefits, and components [49]. A shortened intervention (6 rather than 12 weeks) was planned to be initially delivered as designed and iteratively refined based on caregiver feedback to improve its acceptability.

### Participants

Caregivers were defined as the primary individuals who provide physical, emotional, and/or instrumental support and assistance to individuals with advanced cancer that the latter consider to be like family. For inclusion in the study, caregivers were required to be:

i) 18 years of age or older;
ii) able to speak and read English; and.
iii) involved in the care of a person with advanced cancer (stage 3 or 4 solid tumors) at home [56].

Exclusion criteria were:

i) already participating in regular PA. Caregivers reporting PA for ≥ 30 min on more than three days per week were considered to have established a regular PA pattern despite caregiving demands. The intervention prioritized those who were insufficiently active or experiencing difficulties maintaining regular PA, thereby reducing ceiling effects.
ii) a current mental illness that had been treated with medication for < 8 weeks or for which the caregiver intended to initiate medication during the period of the intervention. These caregivers were excluded to help ensure changes in the outcomes can be more confidently attributed to the PA intervention rather than the initiation of medication. While this study is not evaluating the effects of the intervention on the targeted outcomes, it was important to evaluate the acceptability of the intervention with the same population as will be targeted in a future randomized controlled trial.
iii) any major physical health problem that precludes physical activity as assessed by the Physical Activity Readiness Questionnaire (PAR-Q) during eligibility screening [57].

The study recruited from three organizations in Canada, including an urban, University-affiliated hospital-based cancer care centre (consisting of several outpatient clinics), and two not-for-profit organizations providing free specialized home nursing care and support for families with a member at the end of life. Approval was obtained from the hospital’s Research Ethics Committee. Both home care organizations, not having their own ethics committees, reviewed the proposal and ethics approval and verified appropriateness for their population prior to granting access to their sites. Staff at each of the organizations were asked to identify caregivers who met the inclusion criteria, inform them briefly about the study and give them an information pamphlet. Posters and pamphlets advertising the study were also placed in waiting areas in the cancer care outpatient clinics, and an advertisement containing information about the study was placed in the hospital newsletter. Caregivers who were referred or self-referred to the study and were interested in hearing more were contacted by the first author to fully inform them of the study, further assess eligibility, and answer any questions. Written informed consent was obtained from all participants. (Recruitment process and eligibility flow sheet can be seen in Supplementary Files 1 and 2).

### Intervention

The provisional PA intervention was designed for caregivers of patients with advanced cancer. Informed by a narrative review of the literature related to caregivers and PA in the context of cancer and the Health Promotion Model [48], its goal is to promote caregivers’ motivation for and commitment to engaging in PA, ultimately increasing or maintaining their PA level over time. A detailed description of the proposed PA intervention can be found in Table 1 using the Template for Intervention Description and Replication (TIDieR) guidelines [58]. This 12-week PA intervention is characterized by: (i) motivational interviewing guided by the Health Promotion Model to inform the development of an individualized PA plan; (ii) goal setting and implementation action plan strategies; (iii) self-monitoring; and (iv) weekly telephone calls for ongoing feedback and support. The PA intervention was designed with the intent that ultimately it would be delivered by homecare nurses or other healthcare or community providers who support caregivers on a regular basis. For the purpose of this initial evaluation, the first author was the intervener. She has a degree in physical education and is a registered nurse. Sensitive to the caregiving context, the proposed intervention is designed to be home-based, and the individualized PA plans facilitate caregivers’ engagement in self-selected activities which includes their preference for where the PA takes place.

**Table 1:** Detailed description of provisional intervention

Name	My Ten: A Physical Activity Intervention for Family Caregivers of People with Advanced Cancer	
Why		The intervention aims to promote caregivers’ motivation for and commitment to engaging in physical activity (PA), ultimately increasing or maintaining their PA level over time. PA is an important health-promoting behaviour with physical and psychological benefits that are well-documented.
What		*Motivational Interviewing*: Caregivers are invited to engage in collaborative, goal-oriented communication to explore their motivations for and commitment to engaging in PA as a health-promoting behaviour. Rather than being directive, motivational interviewing emphasizes a person-centered guiding approach, characterized by open-ended questions, reflective listening, affirmations, and summaries to guide the conversation and empower the caregiver. A respectful, non-judgmental, and empathetic environment fostered by the intervener allows the caregiver to feel safe to explore their own motivations for change in an open, honest manner. The motivational interviewing is guided by the *Health Promotion Model* (HPM; Pender et al., 2002), which outlines the behaviour-specific cognitions and affect that can be explored and modified to influence changes in health-promoting behaviour. These factors include perceived benefits, perceived barriers, perceived self-efficacy, activity-related affect, interpersonal influences, and situational influences. *Goal Setting (Individualized PA Plan)*: Goal setting is used as a motivational strategy, with a specific focus on process goals - the specific, actionable steps taken to achieve the larger performance objective of increasing or maintaining PA. Process goals related to engaging in PA (developed as an individualized PA plan) are structured outlining the frequency, intensity, time, and type of PA the caregiver will engage in. This process goal/PA plan includes an implementation action plan to help elucidate when, where, and how the PA will be carried out. *Self-Monitoring*: A simple PA log (pen and paper) is used by caregivers to self-monitor their engagement in PA. Definitions of PA and light, moderate, and vigorous intensities are provided at the top of the log, along with written instructions for how to complete it. A section for the caregiver to record their PA goals and implementation action plan is also included. Below this is a table to record the frequency, intensity, time and type of each bout of PA. *Feedback and Support*: Regular contact with the intervener provides the caregiver with feedback and support to help facilitate their engagement in PA.
Who Provides		The intervention can be delivered by homecare nurses, social workers, or other health or community care providers who support and are in regular contact with caregivers. For this acceptability study, the intervention was delivered by the 1st author. She has a degree in physical education and is a registered nurse.
How		*First Baseline Visit*^*a*^: An initial face-to-face visit is conducted, during which the intervener establishes rapport with the caregiver to facilitate future motivational interviewing. The intervener instructs the caregiver how to monitor their PA using the simple PA log. The caregiver is asked not to change their PA habits for one week and to record their PA daily to establish baseline PA. The caregiver is given hard copies of the PA log (one/week). *Second Baseline Visit*^*a*^: A second face-to-face visit is conducted, during which the caregiver’s PA log from the previous week is reviewed to establish baseline PA. The intervener and caregiver then engage in motivational interviewing, guided by the HPM (Pender et al., 2002), to collaboratively explore the caregiver’s PA-specific cognitions and affect. The information gleaned from the motivational interview is used to inform the development of an individualized PA plan/process goal. The frequency, intensity, time, and type of PA are chosen by the caregiver and tailored to accommodate their needs, preferences, and circumstances. An implementation action plan is developed alongside this to help facilitate the PA plan/goal. The caregiver is asked to engage in their PA plan over the following week and monitor their PA daily using the PA log. *Weekly Contacts*^*b*^: The intervener provides weekly telephone calls for feedback and support. The caregiver reports their PA log data, and the intervener and caregiver assess it against the PA plan/process goal that was set the previous week. The intervener and caregiver engage in motivational interviewing to explore reasons why the caregiver was able or not to achieve their PA plan/goal. Variations from the set plan/goal are discussed to develop alternate strategies. The intervener and caregiver collaboratively re-explore concepts in the HPM as necessary, to inform any modifications to the PA plan/process goal and set a new plan/goal for the coming week. The caregiver is asked to engage in their PA plan over the following week and monitor their PA daily using the PA log.
Where		The first and second baseline visits are conducted at the caregiver’s home or at the hospital during the patient’s visit. The weekly contacts are delivered through telephone calls provided by the intervener.
When and how much		The second baseline visit is conducted one week following the initial baseline visit. Then, 12 consecutive weekly telephone calls are provided by the intervener.^c^
Tailoring		Motivational interviewing is inherently a highly tailored, person-centered approach. The strategies used to guide the conversation (open-ended questions, reflective listening, affirmations, and summaries) are tailored based on the caregiver’s readiness for change, personal values, and PA-specific cognitions and affect. The PA plan/process goal (frequency, intensity, time, and type of PA) and implementation action plan (when, where, and how) for each caregiver is also tailored to their unique needs, preferences, and circumstances based on the information gleaned during motivational interviewing.

^a^ The baseline visits might be combined if a retrospective PA log is considered acceptable to establish baseline PA for the purposes of the intervener

^b^ The weekly contacts could be delivered in person rather than by telephone if it is a week when the intervener is already making a home visit and will have time for the intervention

^c^ For this test of acceptability, only 6 weekly telephone calls were conducted

Two baseline home visits are conducted. The first visit is used to establish rapport between the intervener and the caregiver, and to instruct caregivers how to monitor their usual PA using a simple PA log for one week (Supplementary File 3). This information is used to establish baseline levels of PA. A second home visit is then conducted during which the intervener and caregiver engage in motivational interviewing to collaboratively explore the caregiver’s PA-specific cognitions and affect as outlined in the Health Promotion Model (perceived benefits; perceived barriers; perceived self-efficacy; PA-related affect; interpersonal influences; and situational influences) to inform the development of an individualized PA plan. That is, the frequency, intensity, time, and type of PA, which comprise the plan, are chosen by the caregiver and tailored to accommodate the caregiver’s needs, preferences, and circumstances. The PA plan functions as the PA goal (process goal), including an implementation action plan to achieve the goal. The aim is to engage caregivers in setting realistic plans/goals that are feasible for their individual caregiving situations and gradually increase or maintain their PA over time. PA is measured daily by self-report using a simple PA log. Weekly telephone calls are provided by the intervener for ongoing feedback and support. Caregivers report their PA data during each weekly telephone contact, and the intervener and caregiver collaboratively assess the PA data against the PA goal that had been set and revisit any concepts in the Health Promotion Model and adjust the PA plan, as necessary. Given the primary objective of this initial study was to assess acceptability, an abbreviated 6-week version of the intervention was delivered. Lack of published protocols describing the development of PA interventions provided little guidance for the timeframe for this phase of the study. As such, the length of the intervention for this phase of the research process was derived from the experience of a thesis committee member with expertise in the development of complex interventions for caregivers [59] and the clinical experience of a physiotherapist consulted for their expertise in implementing PA programs for individuals with cancer.

### Data collection

A demographic questionnaire was administered at the start of the first home visit. Recruitment and attrition rates as well as reasons for dropout were tracked.

#### Qualitative data

Each of the intervention delivery time points (except for the first home visit) was audio-recorded and field notes were taken to capture the process of intervention delivery. Throughout the period of the intervention, caregivers were also invited to engage in 6 semi-structured interviews (at the end of each weekly telephone call) to obtain their feedback as they engaged in the intervention [60]. Broad, open-ended questions were used to generate responses that described the acceptability of the structure and process of the PA intervention as well as any modifications that needed to be made. Caregivers were asked to comment broadly on barriers and facilitators to achieving their PA goals in the previous week. The open-ended semi-structured interview guide also included prompts to explore caregivers’ perceptions of each component of the PA intervention (i.e., motivational interviewing, individually tailored PA plan and goal setting, self-monitoring, weekly phone calls for feedback and support) and any suggestions they had to make each element more acceptable. At the end of 6 weeks, caregivers were invited to participate in a follow-up semi-structured interview to discuss the overall intervention appropriateness and usefulness in addressing their self-care needs and its suitability for caregivers in similar contexts. They were also asked to suggest ways to enhance the relevance and usefulness of the PA intervention, and to identify any element that may be missing or need to be modified. In addition, caregivers’ perceptions of the potential benefits of the PA intervention were explored. Before each interview, caregivers were encouraged to provide honest answers as these would be most helpful to understand caregivers’ perspectives and make any necessary changes to the intervention. All the interviews were audio-recorded, and field notes were written. The weekly data collection form, weekly semi-structured interview guide and the follow-up interview guide can be found in Supplementary Files 4–6.

#### PA data

A study-specific self-report PA log was used to record the frequency, intensity, duration, and type of PA completed throughout the intervention. Caregivers reported their PA from the previous week during each of the weekly telephone calls of the intervention. This pragmatic approach enabled assessment of adherence to individualized PA plans, which could not be adequately captured using standardized PA questionnaires [61, 62]. Although self-report logs are subject to recall and social desirability bias, they provide detailed contextual information regarding PA behaviour and are well suited to behavioural intervention studies in which individualized PA plans are evaluated [61, 62].

#### Outcome measures

Outcome measures were not included for efficacy evaluation, but rather to trial the use of the same methods that would be used in a future randomized controlled trial. At the second home visit and week 6, caregivers completed the Positive and Negative Affect Schedule (PANAS; primary outcome) [63], Lee Fatigue Scale (LFS) [64], and a general self-rated health question. Verbal feedback was solicited after the outcome measures were completed at the second home visit, which was captured in the audio recording, and written feedback was solicited at the end of the package of outcome measures at week 6 by asking participants about the acceptability of the measures (i.e., length of time to complete, unclear or upsetting items).

### Data analysis

Data were analyzed to assess the acceptability of the intervention, that is: (i) attrition; (ii) adherence to the intervention and PA plans; (iii) perceived relevance of the intervention; (iv) perceived benefits and usefulness of the intervention; and (v) suggestions for improvement.

**Fig. 1:**
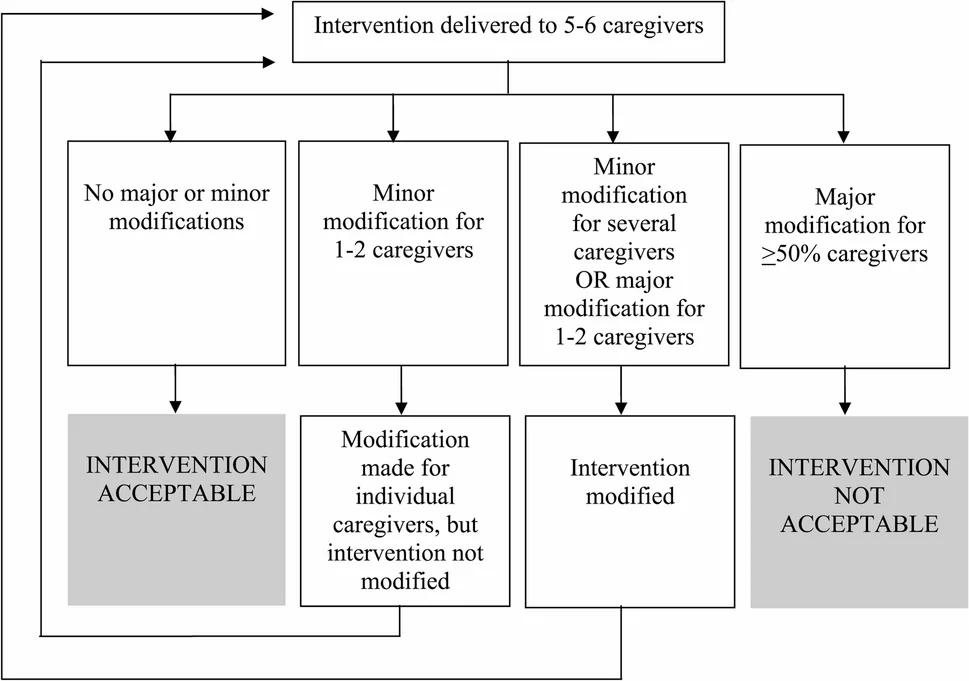
Guidelines for judging acceptability

The guidelines for judging acceptability of the intervention were set a priori (Fig. 1). Suggestions for improvement were conceptualized as being driven by the desire to improve facilitators of adherence, relevance, and usefulness of the intervention. A *major modification* included any revision that would significantly change the structure or process of the intervention, which would make it inconsistent with the underlying theoretical rationale or preclude engagement in the PA intervention. A *minor modification* included any revision that would slightly change the structure or process of the intervention but in so doing the intervention would remain theoretically justified and caregivers would continue to engage in the PA intervention.

#### Attrition and adherence to the intervention and PA plan

Rates of attrition and adherence were calculated and reported as means or frequencies. The rate of adherence to the individual PA plans was determined by calculating how many weeks the participants were able to engage in PA levels at or above their baseline in relation to the number of weeks that they were in the study. PA data were converted to metabolic equivalent of task (MET) hours to account for the duration and intensity of reported PA. MET hours were calculated by multiplying the duration of each activity by its corresponding MET value from the Compendium of Physical Activities and summing across activities completed during the week [61]. The conversion of PA into MET hours is a standardized, internationally accepted scoring procedure based on established metabolic equivalent values and provides a common metric to compare overall PA across activities of varying intensity and PA levels across participants [61].

#### Qualitative data analysis

A hybrid approach of deductive and inductive content analysis was used to provide structure and identify patterns within the data [65–67]. A pre-defined matrix of analysis (deductive approach) was used within which columns represented relevance and usefulness of each of the elements of the intervention (motivational interviewing, individualized PA plan and goal setting, self-monitoring, feedback and support), perceived benefits, and suggestions for improvement. Likewise, columns representing the frequency, intensity, time, and type of PA that caregivers engaged in were included. The use of an inductive approach within the structured, pre-defined coding scheme allowed patterns of codes to emerge in relation to the acceptability of the intervention and the nature of caregivers’ engagement in PA [68, 69].

The audio-recordings from the intervention delivery time points, and weekly and follow-up interviews were transcribed verbatim by a research assistant. The first author listened to each of these recordings in their entirety and double-checked the transcribed documents against the recordings for accuracy. Qualitative data from the transcripts, field notes, and PA logs were reviewed and coded for correspondence with the elements identified in each of the pre-defined columns of the analysis matrix. Any recurring ideas were assigned codes that represented these patterns. Similar codes were then organized into broader categories. Data were analyzed by comparing data within and between participants in an ongoing, iterative process to determine areas of agreement and areas of divergence to ascertain the perceived relevance, usefulness, and benefits of the PA intervention and what adaptations and revisions, if any, needed to be made so that the PA intervention was acceptable to caregivers. The number and nature of any issues related to the outcome measures were discussed with the research team for consideration prior to proceeding with the pilot randomized controlled trial.

### Ensuring rigour

The goal of rigour, or trustworthiness, in qualitative research is to establish confidence in the findings of the study by ensuring the research design and methods are explicit, consistent, and replicable [70]. The methods used to enhance rigour in this study were: (i) engaging in a prolonged period of data collection, (ii) triangulation of various data sources, (iii) review and discussion of emerging findings among the first and third author and, at various points, individual members of the thesis committee, (iv) remaining cognizant of using the participants’ own words throughout data analysis, interpretation and description, (v) ongoing verification of emerging findings with participants, (vi) keeping an audit trail of study activities and decisions, (vii) detailed reporting of methods, (viii) a detailed description of the PA intervention following the TIDieR guidelines [58], and (ix) providing a rich description of the study context [70].

## Results

### Participants

Eighteen caregivers were referred and/or self-referred to the study. Of these, 2 reported being too busy and overwhelmed to participate and 2 felt they did not need support for self-care at the time. In addition, 2 caregivers were already regularly engaged in PA and for 2 others, the patient died before the caregiver was screened for the study. In the end, 10 caregivers were recruited for this study.

A preponderance of participants were men (*n* = 9). The mean age of caregivers was 61 years (range 44–77), and care recipients had a mean age of 63 years (range 39–87). Eight of the caregivers were the spouse of the person they were caring for, one an adult child of the care recipient, and one a friend. All care recipients were living with stage III or IV solid tumors (stage III *n* = 2; stage IV *n* = 8), representing various types of cancer (liver *n* = 1; ovarian *n* = 2; lung *n* = 2; breast *n* = 2; bone *n* = 1; bladder *n* = 1; and oral *n* = 1).

### Attrition

Eight of 10 caregivers completed the 6-week trial implementation of the PA intervention. One caregiver did not wish to continue after the first home visit because the person they were caring for died. Another caregiver dropped out after 2 weeks because they felt they were already active and did not need the support to maintain their level of PA. They stated that they thought the intervention would be good for those who were more sedentary.

### Adherence to intervention

The number and mean length of home visits and weekly telephone calls for intervention delivery is shown in Table 2. Ten first home visits and 9 second home visits were conducted, with a mean duration of 53 (this included time to obtain informed consent) and 58 min respectively. In total, 47 weekly telephone calls were conducted (mean duration = 16 min; this included time for the weekly interviews at the end of each call). Missed weekly telephone calls (*n* = 3; 6%) were due to the caregiver being out of town or unavailable due to a hospital admission of their family member. Despite missing these weekly telephone calls for feedback and support, caregivers were still able to record their PA on their PA log and reported it by leaving a message on the study’s confidential voicemail or reporting it during the next weekly telephone call.

**Table 2:** Intervention delivery time points

Procedure	N	Duration mean (range) in minutes
Baseline home visits		
First visit	10 (8 home; 2 hospital)	53 (30-75)
Second visit	9 (8 home; 1 hospital)	58 (30-150)
Weekly phone calls	47	16 (6-63)

### Adherence to PA plans

The mean rate of adherence to the individual PA plans was 85%. The proportion of caregivers that adhered to the PA plans 76–100% of the time was 89%. When goals were not met it was most often due to immediate competing demands associated with an acute, unexpected crisis with the ill family member or an issue with the caregivers’ own health. In instances of an unexpected crisis with the care recipient, engaging in PA was especially challenging as caregivers chose to put the patient’s needs before their own.

### Relevance of the PA intervention

Overall, the caregivers perceived the PA intervention to be acceptable in the context of their caregiving situation. Working collaboratively with the intervener to explore the concepts in the Health Promotion Model and develop a PA plan, the flexibility of individualized PA plans and setting realistic plans/goals, self-monitoring using a simple PA log, and weekly telephone calls for feedback and support were all components of the intervention that caregivers deemed useful to motivate them and help them engage in and adhere to a PA plan as a health-promoting strategy.

#### Motivational interviewing

Working collaboratively with the intervener to explore the concepts in the Health Promotion Model to inform the development of a PA plan was reported by all the caregivers to be a strength of the PA intervention. Most caregivers reported that co-creating an individualized plan versus being presented with a preset PA plan was important. Partnering with the intervener and playing an active part in developing their own PA plans helped caregivers feel invested in this self-care strategy.

“…it was very helpful to have a program…coming up with things that I know that I can do and that I want to do and I like to do…I wouldn’t have been very good with something if you had said to me ‘well, you really need to do….’ uh, something I really don’t like to do…and I also saw that it was certainly attainable…I think it’s great that it wasn’t just all by telephone, you know, that I met you and we were able to come up with this together…” [Participant A01].

About half of the caregivers emphasized that good interpersonal and empathic communication skills were important attributes for the intervener to have to promote the collaborative relationship and facilitate the process of the intervention.

#### Setting goals/individualized PA plans

All caregivers identified goal setting, in the form of an individualized PA plan (process goal), as an important aspect of the intervention. Characteristics of the individualized PA plans/process goals developed jointly by the intervener and the caregivers are shown in Table 3. At the outset of the study, it was not known how much or what types of PA caregivers of those with advanced care are willing and/or able to do. The strategy of accumulating short bouts of PA throughout the week was embraced by many of the caregivers. Several chose to incorporate some activity into their regular routines, such as taking stairs versus the elevator or parking further away and walking when they did their shopping. However, many individual PA plans/goals also included purposeful ‘time-outs’ to engage in more traditional types of PA, such as strength training, circuit training, and walking outdoors, which were above daily routine activities. These bouts of PA were done in shorter or longer bouts, depending on the context of the caregiver’s schedule in a particular week, and all were done at or near the caregiver’s home.

**Table 3:** Characteristics of individualized PA plans/process goals

Participant	Frequency	Intensity	Time (duration) in minutes	Type
A01	Every other day	Moderate Moderate	45 (morning) 45 (afternoon)	Indoor circuit training *(sit-ups/dancing/arm weights/jumping jacks)* Outdoor walking
A03	Daily Daily Mon-Wed-Fri Tues-Thurs-Sat	Light Moderate Moderate Moderate	10 (morning) 20 15 (evening) 15 (evening)	Stretching Outdoor walking Strengthening exercises - upper body Strengthening exercises - lower body
A04	Mon-Wed-Fri Tues-Thurs-Sat Mon-Sat	Moderate Moderate Light	30 (daytime) 30 (daytime) 20–30 (evening)	Sword training Weight training Stretching
A05	Daily Mon-Wed-Fri	Moderate Light	20–30 (evening) 5–10 (evening)	Dog walking Strengthening exercises – lower body
A06	Daily	Light Moderate	15–20 (morning) 20 (evening)	Back stretches Outdoor walking
A07	3 days per week	Moderate	30	Indoor circuit training *(treadmill/elliptical/abdominal machine/pushups)*
A08	Wed 4 days per week	Moderate Moderate	45 5–10	Outdoor walking Outdoor walking
A09	4 days per week	Light	60 (evening)	Outdoor walking
A10	5 days per week 2 days per week 3 days per week	Moderate Moderate Light	30 15 5	Outdoor walking Outdoor cycling Stretching

*Participant A05 dropped out after week 2

Generally, caregivers were eager to set initial plans/process goals for PA that were quite high, often meeting or exceeding the Canadian PA guidelines of 150 min of moderate-vigorous intensity PA per week. They felt confident that having the option to accumulate short bouts of PA throughout the week was feasible for them to achieve these goals. When caregivers set initial PA goals that were quite high, they often chose to keep these goals throughout most weeks of the study versus gradually increasing them each week as was planned in the initial design of the intervention. Examples of caregiver’s PA plans/process goals over 6 weeks are shown in Table 4.

**Table 4:** Examples of PA plans/process goals over 6 weeks

		Frequency		Intensity	Time (duration) in minutes	Type
Participant A01	Initial PA plan/goal	Every other day		Moderate	45 (morning)	Indoor circuit training *25 sit-ups* *dancing x 2songs* *3 × 10 reps arm weights* *3 × 10 jumping jacks*
				Moderate	45 (afternoon)	Outdoor walking
	Week 1	PA plan/goal remained the same				
	Week 2	PA plan/goal remained the same				
	Week 3	Odd days		Moderate	45 (morning)	Indoor circuit training *40 sit-ups* *dancing x 2songs* *4 × 10 reps arm weights* *3 × 10 jumping jacks*
		Even days		Moderate	45 (afternoon)	Outdoor walking
	Week 4	PA plan/goal remained the same				
	Week 5	Odd days		Moderate	45 (morning)	Indoor circuit training *40 sit-ups* *dancing x 2songs* *4 × 10 reps arm weights* *3 × 10 jumping jacks*
		Even days 1 day/week 1 day/week		Moderate Light Moderate	45 (afternoon) 45 90	Outdoor walking Yoga Hike with a friend
	Week 6	Odd days		Moderate	45 (morning)	Indoor circuit training *40 sit-ups* *dancing x 2songs* *4 × 10 reps arm weights* *3 × 10 jumping jacks*
		Even days 1 day/week 1 day/week		Moderate Light Moderate	45 (afternoon) 45 90	Outdoor walking or bicycling Yoga Hike with a friend
Participant A06	Initial PA plan/goal	Daily		Light Moderate	15–20 (morning) 20 (evening)	Back stretches Outdoor walking
	Week 1	PA plan/goal remained the same				
	Week 2	PA plan/goal remained the same				
	Week 3	PA plan/goal remained the same				
	Week 4	PA plan/goal remained the same				
	Week 5	PA plan/goal remained the same				
	Week 6	PA plan/goal remained the same				
Participant A08	Initial PA plan/goal	Wed 4 days/week	Moderate Moderate		45 5–10	Outdoor walking Outdoor walking
	Week 1	PA plan/goal remained the same				
	Week 2	PA plan/goal remained the same				
	Week 3	PA plan/goal remained the same				
	Week 4	PA plan/goal remained the same				
	Week 5	Wed 4 days/week Daily	Moderate Moderate Moderate		45 5–10 5–10	Outdoor walking Outdoor walking Use stairs vs. elevator
	Week 6	PA plan/goal remained the same				

Caregivers were largely able to meet or exceed their weekly PA goals. Caregivers reported that setting PA plans/goals that were attainable gave them confidence in their ability to reach these, which helped motivate them to continue engaging in their PA plan. Occasionally, the frequency, intensity, time, or type of PA that they engaged in was different than their weekly plan due to various circumstances in their week, but they still achieved the PA goal they were aiming for owing to the flexibility of the PA plan. Even when caregivers did not meet their set weekly PA goals, their level of PA was often still above their baseline PA, aligning with the overall goal of the intervention.

“I was so tired the last couple of weeks, that if I didn’t have the goals, I would have been, you know…better to miss your goal by a little, than it not happen.” [Participant A06].

In weeks when caregivers found it challenging to engage in their PA plan, they frequently began the process of reporting their weekly PA with an apology or statement about not having done as much PA as they had hoped. However, while working through their PA logs and engaging in discussion with the intervener they came to realize that the short bouts of PA had added up to meeting or exceeding their goals. Caregivers expressed feeling proud of the PA they had achieved and reported feeling increasingly confident that setting feasible plans/goals and being able to accumulate short bouts of PA made it possible for them to engage in this health-promoting behaviour whilst being a caregiver.

All caregivers reported that the flexibility of a home-based, individually tailored PA plan made engagement in the PA intervention feasible. The demands of caregiving were time consuming, unpredictable, and altered regular routines for many of the caregivers. In relation to this, caregivers reported that an individualized PA plan/goal where the frequency, intensity, time, and type of PA were tailored to meet their needs, interests, and circumstances helped to address logistical barriers such as lack of time, and made it both enjoyable and feasible to engage in.

“…as we built it, you asked what I like [to do], so it’s based on that. So, it makes it harder to ignore if it’s something that you do like. And also, the way that you incorporate it into the day…I’m not a scheduled person, so I love the flexibility to do it when I feel like it, or when the opportunity arises.” [Participant A04].

Moreover, some caregiving situations necessitated that the caregiver stay near to the patient or not be away from home for significant periods of time. Caregivers expressed that they would not have been able to engage in PA that was strictly scheduled or required them to attend a class. Therefore, having the option to plan bouts of PA at, or near, home was viewed by these caregivers as a positive factor, facilitating engagement in the PA plan and meeting their goal.

“Personally, I did it more because of that [being home-based]…because I would not have taken the time to leave home, to go to X place to do an hour gym. I have no time to waste for that.” [Participant A08].

#### Self-monitoring

Several caregivers reported that the PA log served as a helpful reminder to engage in PA and helped them to see the amount of PA they were able to achieve each week, which added to their confidence and motivation to adhere to their PA plans.

“I always have it [PA log] on my table at home there so I want it to remind me to exercise, so I mark it down…and it’s actually a great motivator because if I don’t think about it, I’m not going to do it.” [Participant A07].

All caregivers reported that the PA logs were easy to use. One caregiver suggested that an online version of the log might make it easier to record their PA and submit the log, but all other caregivers expressed their preference for the pen and paper version of the PA log versus a digital option or some other technology-based approach to tracking PA (e.g., smartwatch). A few of these caregivers commented that they did not want to “learn one more thing” amid the many new demands and responsibilities already being placed on them in their caregiving role.

#### Feedback and support

Whether caregivers had been actively engaged in PA prior to caregiving or PA was a new self-care strategy for them, many caregivers reported that the weekly calls for feedback and support were helpful to assess their PA goals each week, problem solve any barriers, and modify their plan as necessary, finding creative solutions to engage in PA in the context of caregiving where time, motivation, and other factors present specific challenges to self-care. Commonly, caregivers expressed that having the intervener call them weekly simply made them feel important and provided much needed encouragement and motivation for them to take time to engage in self-care.

“I really appreciate it…it was giving me something to look forward to [weekly phone calls]. Like, ‘Ok I did this, this. Oh no, I better do a bit more of that, because Wednesday I’ll be talking to her’…So it gave me motivation…That I really liked, because it makes you feel like you’re important.” [Participant A08].

### Perceived benefits and usefulness

All caregivers reported benefits of engaging in the PA intervention. The most common benefit noted by caregivers was that engaging in PA provided a much-needed break from caregiving responsibilities.

“It [the PA program] was beyond acceptable. Highly recommended to everyone. I think all caregivers should do this…it’s a way to take a break. And we need to take breaks. We can’t be on 24/7. It’s impossible actually.” [Participant A04].

Moreover, many caregivers reported that they felt stronger, physically and psychologically, and were thus better able to care for their family member given these benefits of engaging in PA. The following caregiver described how engaging in PA helped them to decrease stress and tension, increase energy, and generally help them ‘feel better’, which was a common sentiment among caregivers in the study.

“Within a couple of weeks I could feel the benefit…that was encouraging. We went through a bit of a rougher time with [spouse] but I still kept it up [PA] which I believe helped me through that period. I can feel my anxiety levels are somewhat less (…) This [PA program] is a very feasible, realistic way to take care of mental state, and physical, and to build that up in you so you can continue doing what you’re doing.” [Participant A01].

Many caregivers also reported that one of the main benefits of engaging in the PA intervention was feeling confident in their ability to care for themselves whilst being a caregiver. For several caregivers, this was a reminder of the importance of self-care for their own health and wellbeing, and to be able to continue providing care.

“It helps me with energy, and it reminds me that it is important that I do it, take care of myself physically because it is a lot of physical endeavor with my kids and my wife and the pressures, definitely.” [Participant A07].

All the caregivers shared various ways in which their caregiving experiences were stressful and several commented on the lack of available resources to support them in their role. Additionally, many caregivers spoke about the need to take care of oneself but expressed feelings of ambivalence about taking time for themselves amidst the caregiving situation. In relation to this, several caregivers expressed appreciation for a study focused specifically on the caregiver and reported that the intervention was a reminder and/or gave them the permission they needed to engage in self-care.

“…[The PA program] is good to give motivation to do other things and permission to step outside of your caregiving situation…it makes the caregiver more thoughtful about their own health, especially their health with respect to taking care of the patient.” [Participant A09].

### Suggestions for improvements

The only suggestions for improvements to the PA intervention were in relation to the PA log. The initial PA log included one row per day on which to record the PA that was done. Several caregivers suggested that it would be helpful to have more lines to record daily activities on the PA log, particularly if they were accumulating several short bouts of PA throughout the day. In addition, a few of the caregivers suggested that the column to record PA intensity on the log be presented as check boxes for each intensity instead of having to write out the intensity for each bout of PA. These changes were noted as modifications to be made prior to the next phase of the study.

### Outcome measures

There were no major issues reported by caregivers for any of the outcome measures.

## Discussion

The results of this qualitative study provide evidence of the acceptability of the proposed intervention, a novel empirically and theoretically based PA intervention for caregivers of people with advanced cancer. Caregivers deemed the overall structure and process of the PA intervention to be relevant and useful; namely the motivational interviewing facilitated by a collaborative relationship between the caregiver and the intervener, an individually tailored PA plan, a commitment to PA through the PA plan/goal setting and action planning, self-monitoring, and weekly telephone calls for ongoing feedback and support. As per Fig. 1, only minor modifications to the layout of the PA log were suggested by caregivers, which were implemented prior to proceeding with the pilot randomized controlled trial, but aside from this, no modifications to the PA intervention were necessary based on the feedback from caregivers during its trial implementation. Caregivers reported that the intervention facilitated problem solving to overcome barriers and immediate competing demands often present in their caregiving situation, and increased their confidence in their ability to engage in PA. Caregivers also expressed that PA gave them a valuable time out from their caregiving responsibilities to focus on their own health and well-being, which helped them to manage the pressures of caregiving and ‘feel better’, thus enabling them to better care for their family member.

While qualitative interviews provided rich insight into why caregivers found the intervention acceptable, quantitative indicators of acceptability, including a relatively low attrition rate (20%) and high adherence to the individualized PA plans (85%), offered complementary evidence that caregivers can engage in and adhere to a PA plan that considers and is tailored to their individual caregiving situation. With this flexibility, caregivers often chose to engage in shorter, more frequent bouts of PA to accommodate their caregiving circumstances. Derived from the findings, the PA intervention came to be called *My Ten*, to reflect the short breaks that caregivers prioritized to tend to their own health through engagement in PA. The convergence of the qualitative and quantitative findings strengthened the overall assessment of acceptability and provides a strong rationale for proceeding to pilot randomized controlled trial evaluation.

This study contributes to an emerging body of evidence related to the acceptability of PA as a health-promotion strategy among caregivers in the context of cancer [17, 31, 71]. Moreover, only one other health promotion intervention involving PA (C-PRIME) was found that focused exclusively on caregivers of those with advanced cancer [31]. Developing an intervention explicitly for caregivers of those with advanced cancer is important, as the experience and needs of these caregivers is different than those earlier in the cancer trajectory owing to more complex care responsibilities and greater dependency of the patient [32, 33], which increase as the patient’s disease progresses [39]. Another qualitative study examining the acceptability of TEMPO, a 10-week, web-based dyadic intervention, designed to facilitate psychosocial and PA self-management skills among men with prostate cancer (various stages) and their caregivers, reported that PA was the preferred self-management coping strategy chosen by dyads [71]. While the dyads in this study deemed the intervention to be acceptable and helpful overall, feedback emphasized their desire for a more individually tailored approach based on stage of disease, fitness level, and type of support systems available.

Collaboratively developing an individually tailored PA plan/goal with the intervener was an aspect of *My Ten* that caregivers noted made it particularly fitting in the context of their caregiving situation. Specifically, engaging in motivational interviewing guided by the Health Promotion Model [48] allowed the intervener and caregiver to explore the perceived benefits of and barriers to PA, perceived self-efficacy, PA-related affect, interpersonal influences, and situational influences that have been shown to directly or indirectly influence engagement in PA and adherence to PA plans. Consideration of these factors informed the *f*requency, *i*ntensity, *t*ime, and *t*ype, or “FITT” [72], of PA that characterized the individualized PA plan and process goal.

To our knowledge, there are no other studies with cancer caregivers in which the intervener intentionally engages with the caregiver to explore factors known to influence engagement in PA and then uses this information to co-design an individualized PA plan/process goal tailored to caregivers’ interests, abilities, and circumstances. In the C-PRIME intervention [31], health-promoting behaviours, including PA, are monitored using a Fitbit and displayed on a digital platform. Caregivers receive health coaching via 8 weekly telephone calls (15–20 min) during which the health coach covers one of eight different topics related to health promotion, including one on exercise (i.e., exercise guidelines, goal setting) and data from the digital platform are reviewed with the caregiver to set goals related to specific health-promoting behaviours. However, it is not clear whether all caregivers are required to target PA as a health-promoting behaviour and the development of a PA plan to facilitate caregivers’ engagement in PA is not described. TEMPO [27], a dyadic web-based intervention includes a self-directed process of selecting activities where patients and caregivers served as each other’s coaches to develop a PA plan. While this study included dyads representing various stages of cancer, the experience with the intervention was not differentiated between these groups. Individuals living with advanced cancer often experience challenges with engaging in PA [46, 47], owing to worse symptom burden [33, 45] and decreased functional ability compared to those at earlier stages of disease [32, 39], which may also make it difficult for them to enact a coaching role for their caregiver. Other interventions are characterized by more structured center-based group exercise programs where PA plans are devised from a pre-set schedule of exercises and tailored only to caregivers’ fitness levels with the aim of gradually increasing towards a target level of PA [17]. However, most caregivers in the current study reported that attending a gym or scheduled exercise program was not feasible for them in the context of their caregiving situation, again underscoring the relevance of individually tailoring all aspects of the intervention, including where PA occurs.

In addition to individually tailored PA plans/process goals, weekly telephone calls for feedback and support were viewed by caregivers as an essential aspect of *My Ten*, providing a useful reminder of the importance of self-care and motivation to engage in and adhere to PA plans. This reflects an underlying tenet of the Health Promotion Model [48] that ongoing follow-up is necessary to assess if the individually tailored plan is working, provide feedback to make any necessary adjustments, and offer encouragement and support. Other PA interventions for caregivers in the context of cancer have also used feedback and support as an intervention strategy. TEMPO [27] is characterized by caregiver and care recipient dyads engaging in mutual coaching and encouragement as they participate in the intervention. However, most of the caregivers were caring for someone at an earlier stage of cancer where the care recipients’ health status still allowed them to engage in PA and provide reciprocal feedback and support. In RECHARGE [17], a 12-week structured exercise program, caregivers receive feedback from the intervener at the centre where the intervention is delivered as well as social support from other caregivers participating in the group-based exercise classes. The authors did not specify what stage of disease the care recipients were living with but engaging in this intervention requires caregivers to be able to be away from the care recipient and attend center-based exercise classes to receive feedback from the intervener and support from others in the group. While these may be effective approaches to feedback and support in each of these interventions, they may not be feasible for caregivers caring for someone at advanced stages of cancer, where the functional status of the care recipient is declining and care demands and responsibilities on the caregiver are thus often higher [34, 35]. Feedback and support are an important intervention strategy to facilitate engagement in PA [73]. This strategy is a main element of *My Ten*, intentionally operationalized through a collaborative relationship with the intervener to be sensitive to caregiving in the context of advanced cancer.

*My Ten* was designed to minimize barriers to PA while fostering the collaborative relationships and ongoing support identified as important determinants of health-promoting behaviour [48]. Delivering the intervention in caregivers’ homes through a combination of home visits and weekly telephone calls accommodated the competing demands of caregiving by reducing the need for travel and allowing participants to engage in PA within their usual environment while staying near the care recipient [74]. This blended model is consistent with current evidence supporting nurse-delivered home-based and telehealth interventions as accessible and effective approaches to support patients with advanced cancer and their caregivers [13, 75]. Although telehealth has become an increasingly accepted mode of intervention delivery, variability in digital literacy and access to reliable technology remains an important consideration when designing and implementing interventions [76, 77], and face-to-face interactions remain valuable for establishing rapport, facilitating non-verbal communication, understanding caregivers’ unique caregiving contexts, and collaboratively developing an individualized PA plan using motivational interviewing [78]. Incorporating initial home visits also acknowledges that many caregivers value some degree of in-person contact [13, 79]. Consequently, hybrid models that combine an initial in-person visit with remote follow-up may offer the advantages of both approaches (13). In many jurisdictions (including throughout Canada), home care nurses routinely provide ongoing assessment, education and supportive care through a combination of home visits and telephone follow-up. Their role aligns well with the collaborative, person-centered approach required to deliver motivational interviewing and tailored PA support [80, 81]. Designing an intervention to fit within existing models of care may improve its feasibility and future implementation.

An unexpected finding was that 90% of participants in this acceptability study were male caregivers. Although this limits the transferability of the findings to female caregivers, it also addresses an important gap in the caregiving literature. Caregiver intervention research has historically recruited predominantly female participants, and recent reviews continue to identify male caregivers as an underrepresented population in cancer caregiving research, with limited evidence regarding interventions tailored to their needs [82, 83]. At the same time, there is growing recognition that male caregivers may engage with support differently than female caregivers, often favouring practical, problem-focused approaches that emphasize maintaining function and fulfilling the caregiving role rather than traditional psychosocial support alone [83]. Within this context, the high representation of male caregivers in the present study suggests that a tailored PA intervention may represent an acceptable and promising strategy for engaging a group that has historically been underrepresented in caregiver intervention research. While these findings should not be interpreted as evidence that PA interventions are inherently more acceptable to male than female caregivers, they do highlight the potential of health promotion interventions to engage an underserved subgroup of caregivers and underscore the importance of examining the acceptability of *My Ten* across genders in future research.

There are some limitations to the study. Almost all caregivers were men, and it is unknown if women caregivers would perceive the intervention differently. Further testing is needed. In addition, the findings from the current study are based on a small number of caregivers and the intervention was delivered by one individual, which limits variability in intervention delivery parameters. The study was also only conducted in one city; and in the minority language for that setting owing to limitations in the primary investigator’s ability to speak the majority language. Caregivers were interviewed by the primary investigator which may have also created some response bias in favor of the intervention’s acceptability, as there were no critiques about any aspect of the intervention nor suggestions for improvements aside from minor changes to the format of the PA log. Attempts were made to have this interfere as little as possible by instructing participants that honest perspectives about the intervention would be the most helpful.

The absence of objective monitoring of PA and the individualized nature of the PA plan have important implications for the design and evaluation of a future full randomized controlled trial. Although the behaviour change strategies underpinning the intervention, including motivational interviewing, individualized tailoring, goal setting, self-monitoring, and ongoing feedback and support, were delivered in a standardized manner, the resulting ‘dose’ of PA was intentionally individualized to accommodate caregivers’ preferences, functional abilities, and unique caregiving circumstances. Consequently, caregivers engaged in different types, frequencies, intensities, and durations of PA. While this variability reflects the caregiver-centred nature of *My Ten* and is consistent with current recommendations for tailored PA behaviour change interventions [81], it may increase heterogeneity in the behavioural response (PA) and reduce the precision with which intervention effects can be estimated. Incorporating objective measures of PA, such as accelerometers or validated wearable activity monitors, to complement self-reported measures [84], if tolerated by the caregivers, and process measures that assess intervention fidelity and participant engagement [85] should be considered when planning a future full randomized controlled trial. These approaches would facilitate evaluation of whether the standardized behaviour change strategies were delivered as intended, provide a more precise assessment of changes in PA behaviour, and enable exploration of the relationship between the behavioural response and intervention outcomes while preserving the flexibility necessary for individualized PA plans.

## Conclusions

The *My Ten* PA intervention is acceptable to caregivers of people with advanced cancer, helping them to engage in and adhere to their PA plans with no major modifications necessary. Caregivers identified the individually tailored PA plan, motivational interviewing, and feedback and support as central features contributing to the intervention’s acceptability, highlighting the importance of personalization, collaboration, and ongoing support in facilitating engagement in PA. Rigorous development of a novel, evidence-based intervention that is acceptable to caregivers provides an empirical foundation from which to proceed to pilot testing and efficacy studies with the aim of addressing caregivers’ self-care needs, potentially enabling them to maintain their own health and wellbeing and provide care at home for a longer period should they desire to do so. *My Ten* offers another approach to the growing body of evidence related to engaging caregivers in PA as a health-promoting behaviour; one that may be particularly relevant for those in an advanced cancer context. Future research should continue to examine the acceptability and feasibility of *My Ten* in a broader caregiver population. In addition, while the use of a single interventionist and interviewer was appropriate for this exploratory stage of intervention development, allowing consistency in intervention delivery and in-depth exploration of caregivers’ experiences, these methodological approaches should be considered when interpreting the findings and may warrant consideration as *My Ten* progresses toward larger-scale evaluation.

## Supplementary Information

Supplementary Material 1.

Supplementary Material 2.

Supplementary Material 3.

Supplementary Material 4.

Supplementary Material 5.

Supplementary Material 6.

## Data Availability

Given the small number of participants in this study, the datasets generated and/or analyzed during the current study cannot be provided to protect anonymity. Quotes supporting each theme are included in this published article. Additional quotes are available from the corresponding author on reasonable request.

## Abbreviations

PA: Physical activity
MET: Metabolic Equivalent of Tasks
