# Supplementary material for: Acceptability of My Ten: a physical activity intervention for family caregivers of people with advanced cancer
Source: BMC Palliat Care. 2026 Sep 14;25:287. doi: 10.1186/s12904-026-02309-4 (PMC13628949; doi:10.1186/s12904-026-02309-4)
Supplement: Supplementary file 1 [file 12904_2026_2309_MOESM1_ESM.docx]

**Supplementary File 1 - Recruitment Procedure**

Information Sessions Conducted

JGH Oncology Outpatient Clinics/CNR Clinic/

Hope & Cope/Supportive Care Team

NOVA Montreal/West Island

FC not interested in pilot RCT

Not Recruited

Consent &

Participation in Study

Home Visit

FC identified in clinics
and/or community

FC interested in hearing more about pilot RCT
Referrals from nurses/health care team

Investigator calls FC or RA meets with FC

Briefly inform about study

Assess eligibility

Answer any questions

FC not eligible for study

Posters/pamphlets in clinics

Ad in JGH newsletter–

Self-referrals

FC calls investigator

Briefly inform about study

Assess eligibility

Answer any questions

FC not interested in pilot RCT

FC eligible &

interested in study
