# Supplementary material for: Acceptability of My Ten: a physical activity intervention for family caregivers of people with advanced cancer
Source: BMC Palliat Care. 2026 Sep 14;25:287. doi: 10.1186/s12904-026-02309-4 (PMC13628949; doi:10.1186/s12904-026-02309-4)
Supplement: Supplementary file 2 [file 12904_2026_2309_MOESM2_ESM.docx]

**Supplementary File 2 – Study Eligibility Flow Chart**

≥ 18 YEARS OF AGE	NO	INELIGIBLE

YES

ABLE TO SPEAK & READ ENGLISH	NO	INELIGIBLE

YES

INVOLVED IN CARE OF A PERSON WITH STAGE III OR IV CANCER

NO	INELIGIBLE

YES

PARTICIPATING IN PA > 3 DAYS/WEEK AND 30 MIN OR MORE/SESSION

YES	INELIGIBLE

NO

CURRENT MENTAL ILLNESS:

Do you currently have any of the following:

Mood disorder (e.g. depression, bipolar disorder)

Anxiety disorder (e.g. phobia, OCD, panic disorder, generalized anxiety disorder)

NO	YES

Are you currently being treated for this diagnosis?

NO	YES

Do you have intentions	How long have you been on

of starting medication	your most current treatment?

during the study?	Reassess

NO	YES	>8 weeks	<8 weeks	eligibility	8 weeks	after start of

medication

if caregiver

willing

MAJOR PHYSICAL HEALTH PROBLEM AS ASSESSED BY PAR-Q:

≤ 69 YEARS OF AGE	>69 YEARS OF AGE

AND NO TO ALL	OR YES TO ONE OR MORE

CHECK WITH FAMILY DOCTOR:

APPROVAL	NO APPROVAL	INELIGIBLE

**PARTICIPANT ELIGIBLE FOR STUDY**
