# Supplementary material for: Acceptability of My Ten: a physical activity intervention for family caregivers of people with advanced cancer
Source: BMC Palliat Care. 2026 Sep 14;25:287. doi: 10.1186/s12904-026-02309-4 (PMC13628949; doi:10.1186/s12904-026-02309-4)
Supplement: Supplementary file 3 [file 12904_2026_2309_MOESM3_ESM.docx]

**Supplementary File 3 – PA Log**

**PHYSICAL ACTIVITY LOG**

This physical activity log is meant to capture any purposeful bouts of physical activity that you engage in for the purpose of taking care of yourself. These are activities that you do above your daily routine or caregiving activities.

Please record your physical activity daily by marking down the date, the type of activity that you did, how long you did it, and how intense it was. Use a separate line for each bout of physical activity that you do.

*Light-intensity* activities will not cause you to break a sweat or notice a change in your breathing pattern.

*Moderate-intensity* activities will cause you to develop a light sweat and breathe more quickly.

*Vigorous-intensity* activities will cause you to sweat and be 'out of breath'.

| DATE: |  |
| --- | --- |
| GOAL: |  |
| PLAN: | What I will do:  Where I will do it:  When I will do it: |

| **Date** | **Activity**  **(type of activity)** | **Duration**  **(minutes)** | **Intensity**  **(light, moderate, vigorous)** |
| --- | --- | --- | --- |
| *Example:*  Dec 4, 2025 | Walking outdoors | 20 minutes | moderate |
|  |  |  |  |
|  |  |  |  |
|  |  |  |  |
|  |  |  |  |
|  |  |  |  |
|  |  |  |  |
|  |  |  |  |
