# Supplementary material for: Acceptability of My Ten: a physical activity intervention for family caregivers of people with advanced cancer
Source: BMC Palliat Care. 2026 Sep 14;25:287. doi: 10.1186/s12904-026-02309-4 (PMC13628949; doi:10.1186/s12904-026-02309-4)
Supplement: Supplementary file 4 [file 12904_2026_2309_MOESM4_ESM.docx]

**Supplementary File 4 – Weekly Data Collection Form (Telephone Contact)**

PARTICIPANT #:

DATE:

TIME:

LENGTH OF PHONE CALL:

WEEK #:

**PA LOG DATA**

| **Date** | **Activity**  **(type of activity)** | **Duration**  **(minutes)** | **Intensity**  **(light, moderate, vigorous)** |
| --- | --- | --- | --- |
|  |  |  |  |
|  |  |  |  |
|  |  |  |  |
|  |  |  |  |
|  |  |  |  |
|  |  |  |  |

**Assessment of PA goals:**

**MET hours/week:**

| MET hour goal for this week (see Intervener PA Work Sheet) |  |
| --- | --- |
| MET hours this week |  |
| PA goal achieved | YES NO |

- Collaboratively explore concepts in the Health Promotion Model with the family caregiver reflecting on discussion from previous week to develop individualized PA plan (use Intervention Manual and Intervener’s PA Work Sheet to facilitate this process)
- Assist the caregiver to set realistic goals and develop an implementation action plan for the following week
- Reminder to complete questionnaire package **(Week 6 only)**

**Investigator/RA Field Notes re: Telephone Call**

(e.g. Description of caregiver’s engagement in the process of developing the PA plan, setting realistic goals, and developing an appropriate implementation action plan, description of caregiver’s tone of voice,)
