# Supplementary material for: Acceptability of My Ten: a physical activity intervention for family caregivers of people with advanced cancer
Source: BMC Palliat Care. 2026 Sep 14;25:287. doi: 10.1186/s12904-026-02309-4 (PMC13628949; doi:10.1186/s12904-026-02309-4)
Supplement: Supplementary file 5 [file 12904_2026_2309_MOESM5_ESM.docx]

**Supplementary File 5 – Weekly Interview Guide**

*The following questions are to be used for Week 1 Telephone Contact. For weeks 2-6 the researcher should explore these questions taking into account the participant’s answers from the previous week and adapting the questions accordingly.*

I am going to ask you some questions about your experience with the physical activity program. Honest answers will be the most helpful. If there are any problems with the physical activity program we want to know about them because the goal of this part of the study is to revise the physical activity program until it is acceptable to you.

What things helped you to achieve your physical activity goals?

What things made it difficult to achieve your physical activity goals?

Can you please tell me about your experience of developing a physical activity plan with me/the researcher?

What did you find most helpful during the process of developing the physical activity plan?

What was difficult about the process of developing the physical activity plan?

What suggestions do you have to make the process of developing the physical activity plan more acceptable?

Can you please tell me about your experience of setting physical activity goals?

What did you find most helpful about setting physical activity goals?

What was difficult about setting physical activity goals?

What suggestions do you have to make the process of setting physical activity goals more acceptable?

Can you please tell me about your experience of developing an action plan to achieve your goals?

What did you find most helpful about developing an action plan?

What was difficult about developing an action plan?

What suggestions do you have to make the process of developing an action plan more acceptable?

Do you have any additional comments or suggestions about the PA program or the process of developing the PA plan?

Last time we spoke about your experience of using the Physical Activity Log. Do you have any further suggestions about how to make it more acceptable to use?
