# Supplementary material for: Acceptability of My Ten: a physical activity intervention for family caregivers of people with advanced cancer
Source: BMC Palliat Care. 2026 Sep 14;25:287. doi: 10.1186/s12904-026-02309-4 (PMC13628949; doi:10.1186/s12904-026-02309-4)
Supplement: Supplementary file 6 [file 12904_2026_2309_MOESM6_ESM.docx]

**Supplementary File 6 – Follow-up Interview Guide**

Can you please tell me about the effects that this physical activity program has had on you?

*Additional questions/probes:*

Do you recognize any effects on how you are feeling emotionally?

Do you recognize any effects on your level of fatigue?

Do you recognize any effects on your overall health?

Do you recognize any effects of this physical activity program in other areas of your life?

Overall, was the physical activity program acceptable? Why or why not?

*Additional questions/probes:*

What was the most helpful about the physical activity program?

What was difficult about the physical activity program?

What was helpful and/or difficult about having a physical activity program based out of home?

What was helpful and/or difficult about receiving feedback and support to help you develop a physical activity plan?

What was helpful and/or difficult about receiving feedback and support by telephone?

Would you have preferred a different way to receive feedback and support?

What was helpful and/or difficult about having a physical activity program tailored to your needs and/or circumstances?

What was helpful and/or difficult about setting physical activity goals each week?

What was helpful and/or difficult about developing an action plan to achieve your goals?

Can you please tell me about any benefits that you have experienced from this physical activity program?

Can you please tell me about any ways in which this physical activity program may have been harmful to you?

Do you have any further comments or suggestions about how to make the physical activity program more acceptable?

Overall, was the Physical Activity Log acceptable? Why or why not?

Do you have any further comments or suggestions about how to make it more acceptable?

Overall, were the questionnaires acceptable? Why or why not?

*Exploration of any items that were identified as unclear or upsetting.*

Would you have preferred another method of completing the questionnaires besides paper and pencil?

Do you have any further comments or suggestions about how to make the questionnaires more acceptable?

What have you learned through your experience with this physical activity program?

What would you tell other family caregivers who may find themselves in a similar experience?

Would you recommend this type of physical activity program to other people in a similar situation?
